# The fungus *Ustilago maydis *and humans share disease-related proteins that are not found in *Saccharomyces cerevisiae*

**DOI:** 10.1186/1471-2164-8-473

**Published:** 2007-12-20

**Authors:** Martin Münsterkötter, Gero Steinberg

**Affiliations:** 1 Munich Information Center for Protein Sequences/Institute of Bioinformatics and Systems Biology, Helmholz Zentrum München, Germany; 2 Max-Planck-Institut für terrestrische Mikrobiologie, Marburg, Germany; 3 School of Bioscience, Stocker Rd., Exeter University, EX4 4QD, UK

## Abstract

**Background:**

The corn smut fungus *Ustilago maydis *is a well-established model system for molecular phytopathology. In addition, it recently became evident that *U. maydis *and humans share proteins and cellular processes that are not found in the standard fungal model *Saccharomyces cerevisiae*. This prompted us to do a comparative analysis of the predicted proteome of *U. maydis*, *S. cerevisiae *and humans.

**Results:**

At a cut off at 20% identity over protein length, all three organisms share 1738 proteins, whereas both fungi share only 541 conserved proteins. Despite the evolutionary distance between *U. maydis *and humans, 777 proteins were shared. When applying a more stringent criterion (≥ 20% identity with a homologue in one organism over at least 50 amino acids and ≥ 10% less in the other organism), we found 681 proteins for the comparison of *U. maydis *and humans, whereas the both fungi share only 622 fungal specific proteins. Finally, we found that *S. cerevisiae *and humans shared 312 proteins. In the *U. maydis *to *H. sapiens *homology set 454 proteins are functionally classified and 42 proteins are related to serious human diseases. However, a large portion of 222 proteins are of unknown function.

**Conclusion:**

The fungus *U. maydis *has a long history of being a model system for understanding DNA recombination and repair, as well as molecular plant pathology. The identification of functionally un-characterized genes that are conserved in humans and *U. maydis *opens the door for experimental work, which promises new insight in the cell biology of the mammalian cell.

## Background

Fungi are a remarkably successful group of eukaryotes that play an essential part in our ecosystem as symbionts and decomposers of organic material [1,2]. On the other hand, numerous fungi are devastating human and plant pathogens that are a serious threat to agricultural industry and human health [3,4]. In addition, some fungi serve as simple eukaryotic model systems for basic cell biology questions, as they are closely related to animal cells [5,6] and share important cellular processes. In this respect, the most prominent fungal model system is the budding yeast *Saccharomyces cerevisiae*. Its genomic sequence was among the first published in 1996 [7] and more than 77% of the ~6100 genes are assigned to cellular functions [8]. However, this powerful model has its limitations, because certain basic processes found in animal cells, such as long-distance transport along the microtubule cytoskeleton or the removal of the nuclear envelope in mitosis do not exist in budding yeast [9,10]. In recent years, large scale sequencing projects were launched in order to obtain genome sequences from over 80 additional fungi [11]. Among the recently released genomes is that of the basidiomycete *U. maydis *[12], which is also known as a smut fungus on corn. Beside its pathogenic lifestyle and numerous technical advantages of this fungus, the recently published manually annotated proteome, which is available on the public server of the Munich Information Center for Protein Sequences (MIPS; [13,14]) established this fungus as a powerful model system for molecular phytopathology [15-17]. However, *U. maydis *also has a long standing history as a cell biological model system and important basic concepts, such as the molecular mechanism of DNA recombination (e.g. the Holiday Junction was initially described in this fungus [18,19]).

Recently, the importance of *U. maydis *as a model system increased, as studies on the microtubule cytoskeleton in polar growth and mitosis revealed that important processes are conserved between *U. maydis *and mammalians. Such processes are not found in the model fungus *S. cerevisiae *[20]. Among these are kinesin-1- and kinesin-3-based transport processes [21-23], both of which motors are not found in the budding yeast. Another striking example is the removal of the nuclear envelope in mitosis. In contrast to budding yeast, the nuclear envelope is removed at the onset of mitosis in humans [24,25] and in *U. maydis *[26]. Furthermore, in both organisms this is accompanied by the disassembly of the nuclear pores and the recruitment of some pore components to the mitotic chromosomes [27-29]. Interestingly, the mechanistically parallels are reflected by unexpected high sequence conservation of pore components [27]. This strongly suggests that sequence conservation between humans and *U. maydis *coincides with functional similarity. These data indicate that *U. maydis *and mammalian cells share common cellular processes and the underlying molecular machinery that are not found in *S. cerevisiae*. In order to investigate this further, we made use of the SIMAP (Similarity Matrix of Proteins) database, which is based on a Smith-Waterman pair-wise comparison of all known predicted protein sequences available [30]. Using this bioinformatic resource we analyzed the manually annotated proteome set of *U. maydis *and *S. cerevisiae *and the currently accessible protein information of *Homo sapiens*. Surprisingly, we found that the proteome of *U. maydis *is more closely related to humans than to the fungal cousin *S. cerevisiae*. Using the FunCat database that summarizes predicted protein function [31], we demonstrate that many proteins conserved in *H. sapiens *and *U. maydis *can be assigned to certain cellular process. However, a large portion of these proteins are of unknown function. This indicates that essential, yet undiscovered processes are conserved between *U. maydis *and humans.

## Results and Discussion

**Figure 1:**
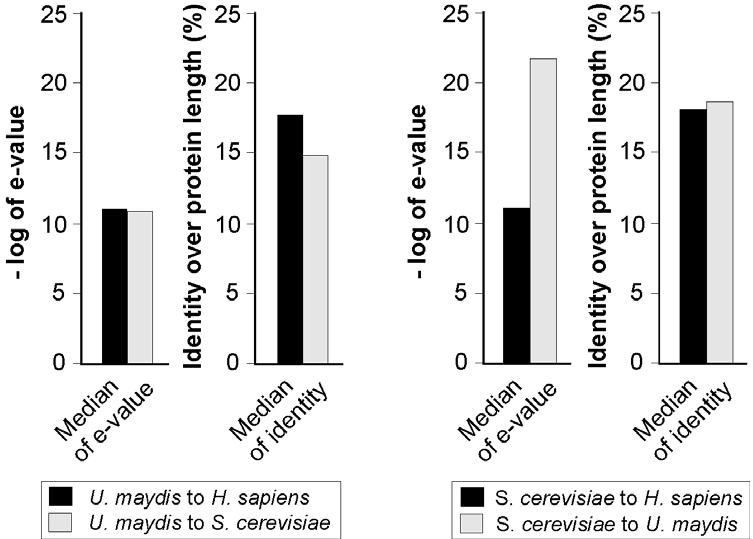
Global comparison of the predicted proteome sequence of the fungi *U. maydis*, *S. cerevisiae *and *H. sapiens*. Based on the Smith-Waterman comparison of all proteins from genome A against all proteins of genomes B the median e-value and the median %-identity value were calculated. Note that *U. maydis *is more similar to humans than to its fungal cousin, whereas the proteome of *S. cerevisiae *is more closely to that of the corn smut.

In a first step we compared general sequence characteristics in coding regions of *U. maydis*, *S. cerevisiae *and *H. sapiens *using the fungal MIPS GenRE databases [32] and human data from the Ensembl database [33]. This included standard parameters, such as the average gene density or gene size (Table 1). In most of the analyzed parameters, including exon size, percent of coding region and average gene density, both fungi are closely related. Next, we did pair-wise comparisons of the whole proteome of *U. maydis *against *S. cerevisiae *and *H. sapiens*. For that a Java client stand-alone application was developed that was used to access the SIMAP retrieval layer (see Material and Methods; [30]). In this analysis, similarity in pair-wise comparison of proteins was indicated by the "e-value" or "% identity over the length of the protein" and the median of these analyses was calculated. Surprisingly, we found that both median values were higher in the *U. maydis*-to-*H. sapiens *set compared to the *U. maydis*-to-*S. cerevisiae *analysis (Figure 1A). In contrast, when the proteome of *S. cerevisiae *was compared with *U. maydis *and *H. sapiens*, the expected outcome was that both fungi are more closely related than the budding yeast to humans (Figure 1B). These results added further support to our initial assumption that *U. maydis *contains additional proteins that are highly conserved to humans, but that are absent from the fungal cousin *S. cerevisiae*.

**Table 1:** General characteristics of the coding region

		***S. cerevisiae***	***U. maydis***	***H. sapiens***
Percent coding	(%)	73.09	60.97	1.22 -2.33
GC content	(%)	39.58	56.26	52.10
Average gene size	(bp)	1451	1747	1526
Average gene density	(kb/gene)	1.99	2.86	125.43
Protein-coding genes		6127	6867	22762^¶^
Exons		6482	9932	267941
Average exon size	(bp)	1372	1208	297
Exons per gene		1.058	1.45	6.149
Genes with introns	(%)	4.81	27.73	97.96

^¶ ^the predicted number increases to 43576 when including splice variants.

**Figure 2:**
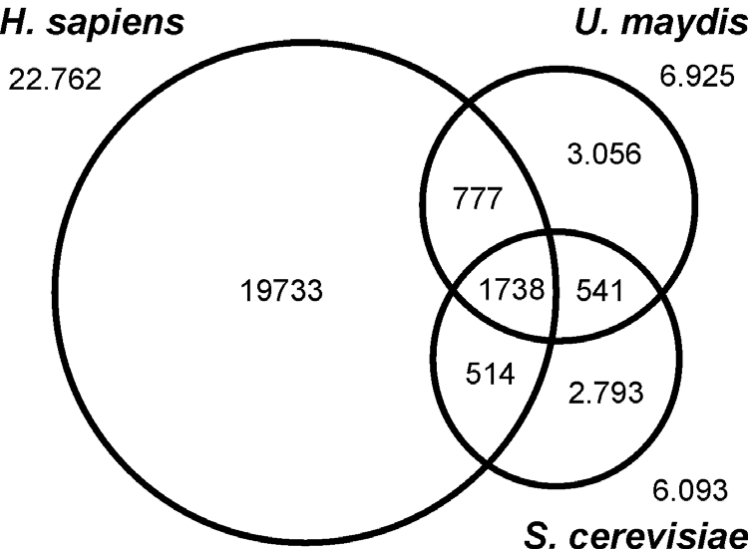
Comparison of the proteome of *U. maydis*, *S. cerevisiae *and *H. sapiens *using cut-off criteria of 20 % identity over the total protein length.

**Figure 3:**
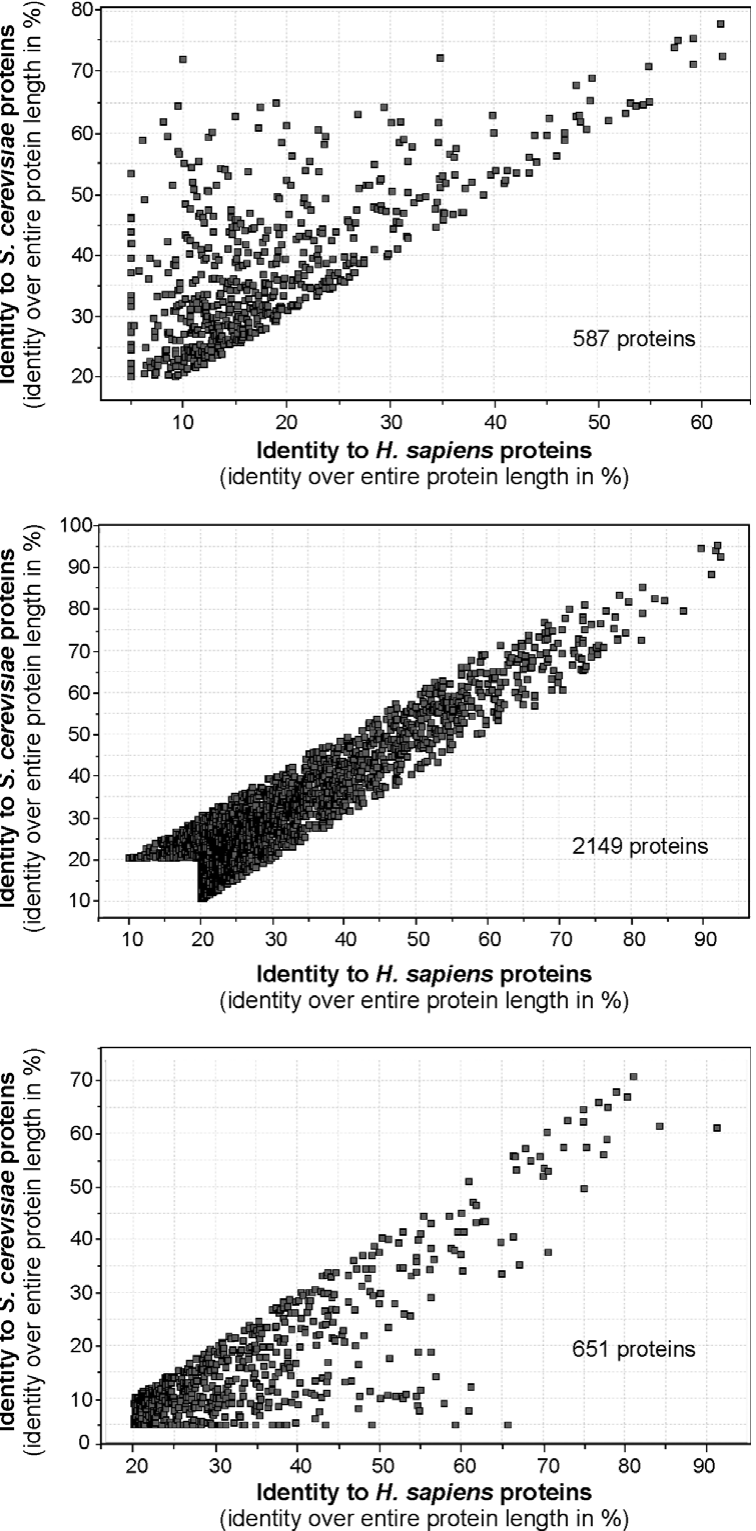
Genome-wide protein homology correlation of *U. maydis against humans and S. cerevisiae *using the % identity over entire protein length. Depicted are predicted *U. maydis *proteins that show > 20% identity to one partner and > 10% less identity to the other. Most proteins (2159) are conserved at similar levels in *S. cerevisiae *and *H. sapiens *(B). However, 587 proteins are more closely related to *S. cerevisiae *(A), whereas an even larger number of 651 are more similar to humans (C). Note that this analysis show "% identity over the total protein length" and thus does not include proteins that share sequence similarity only within a domain.

Its general assumed that sequence conservation in proteins is a consequence of a similar function. However, even unrelated sequences show a certain degree of similarity, which increases in structural motifs such as coiled-coil domains that are found in ~20% of all proteins in *S. cerevisiae *[34]. In order to determine the level of random sequence similarity, we compared unrelated proteins, including TBP (um10143), Myosin I (um11115) and FER1 (um00105) of *U. maydis *against each other. This analysis indicated that random sequence conservation reaches 1–5 % identity. Next, we selected functionally unrelated proteins that contain coiled-coil regions (Table 2). A comparison of these proteins against each other revealed up to 16.9% sequence identity in those proteins that contained extended coiled-coil stretches (Yup1, Tpm2, Kin1 and Clip1; Table 2). Consequently, we considered only protein identity that was 20% or higher as indication for functional conservation, a value that corresponds with previous reports that conserved protein function is indicated by at least 18–20% protein sequence identity [35]. Following this criterion we redid our analysis and found that around 1738 proteins are shared by all three organisms (Figure 2), which represents 25–28% of the proteome of both fungi. Surprisingly, both fungi share 541 proteins, suggesting that only a minor portion (8–9%) of the proteome is fungal specific. In contrast, *U. maydis *has 777 proteins that are more conserved in humans than in budding yeast, whereas *S. cerevisiae *shares only 514 proteins with *H. sapiens*. This again indicates that *U. maydis *is more closely related to humans than to its fungal cousin. However, a strict cut off at 20% could be misleading, as slight differences in sequence conservation around this border will not be recognized. In other words, a protein that has 20.1% identity between both fungi and 19.8% identity in humans would be considered as fungal specific. Moreover, a protein that shares 80% identity between both fungi, but only 20.1% with human will also not be identified. In order to cope with these problems, we included a more stringent criterion for our analysis and considered only those proteins that share ≥ 20% identity in two organisms, but found at ≥ 10% identity in the third partner. In this approach *U. maydis *still shares 587 fungal specific proteins with *S. cerevisiae*, but it has an even larger set of 651 proteins in common with *H. sapiens *(Figure 3). On the other hand, only 287 proteins of *S. cerevisiae *had a conserved counterpart in the human genome (not shown).

**Table 2:** Analysis of coiled-coil domain containing representatives from *U. maydis*

**MIPS ID**	**Gene**	**Function**	**Position**	**% identity**^¶^
um03791	Ums2	Heat shock 70 kd protein	507–539	3.8–7.4
um05406	Yup1	t-SNARE	249–286	3.2–14.1
um11985	Tpm2	Tropomyosin	1–76, 7–161	3.1–13
um04372	Dyn2	Dynein heavy chain	89–156, 311–387, 663–699	3.6–8.5
um04555	Myo5	Myosin V	958–1014, 1022–1078, 1079–1116	3.5–11.3
um06338	Clip1	Microtubule binding protein	561–607, 634–660, 661–708, 709–761, 822–867, 868–941, 1041–1082	10.0–16.9
um04218	Kin1	Motor protein	346–378, 441–551, 604–675, 688–736, 807–873, 874–905	9.4–14.3

^¶ ^% identity over entire protein length to unrelated coiled-coil proteins

**Figure 4:**
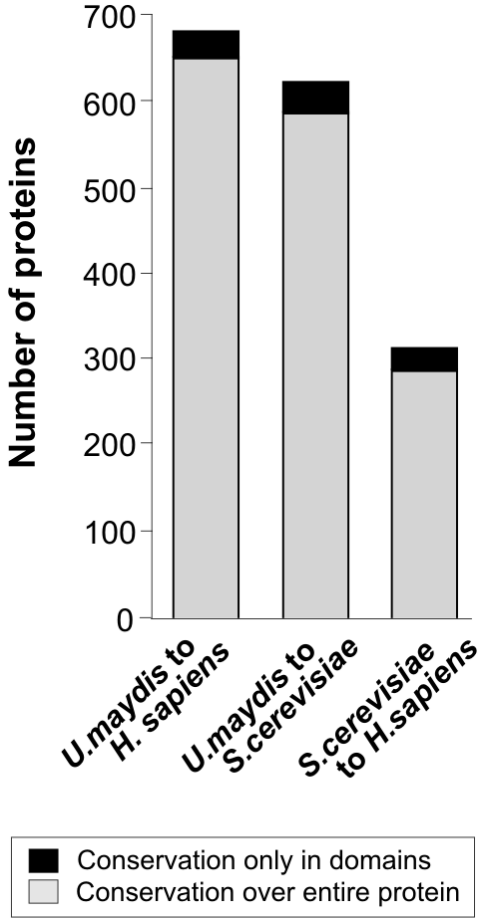
Total number of predicted proteins that show significant similarity (> 20% identity to one partner and > 10% less identity to the other) over their entire length (grey bar) or within domains of at least 50 amino acids (black bar).

The results presented so far were based on a 20% identity over the total protein length. However, many proteins perform specific functions at short domains and functional orthologues might be overlooked when comparing whole proteins. A striking example is the microtubule plus-end binding protein Clip1 in *U. maydis*. It was shown that this protein binds to microtubule plus-ends and contains a CAP-Glu domain and two zinc finger domains [22]. All these features are also typical for the human orthologue CLIP170 [36,37]. However, the overall sequence identity between Clip1 and CLIP170 is only at 19.5%, and significant sequence conservation is only found in the CAP-Glu domain (aa 221–266; prosite motif analysed with ProfileScan; [38]; 50% identity). Therefore, we considered it likely that our analysis has not covered all functional orthologues between the three organisms. Thus, we extended our analysis and compared sequences at a lower limit of 50 aa sequence overlap, which would cover short domains such as the FHA-domain in kinesin-3 (57 aa, PFAM) or the mentioned CAP-Glu domain in Clip1 (65 aa, PFAM) and applied the same criterion as before (≥ 20% identity between two organisms in the overlapping region and 10% less in the third partner). This approach identified an additional 30 proteins being conserved between *U. maydis *and humans (now including Clip1), and 35 in case of a comparison of *U. maydis *and *S. cerevisiae*. The number of proteins conserved between humans and budding yeast increased by 25 additional proteins (Figure 4).

Taken both analyses together our bioinformatic approach revealed 681 homologues between *U. maydis *and humans. In order to better define the orthology relationship among these genes we next performed a best bidirectional hit analysis assuming that orthologous proteins would identify the partner when searching in both directions. Making use of the SIMAP database we found that 620 proteins (~91% of *U. maydis*-to-*H. sapiens *set) fulfil this criterion (see Additional file 1). Thus, our data suggest that ~10% of the *U. maydis *proteins have a role in cellular processes that are most likely conserved in the human cell. On the other hand, budding yeast and humans share 312 unique proteins and yeast and *U. maydis *have 622 proteins in common (Figure 4). The most obvious next question therefore was whether theses proteins can be grouped in functional clusters. In order to address this question, we made use of the Functional Catalogue DataBase (FunCatDB; [39]) that summarizes functional annotation for proteomes from different organisms by assigning proteins of interest to certain cellular processes. In addition, our method provides a p-value that correlates the presence of proteins in the set to the expected abundance of proteins in the same functional groups in the proteome. We first analyzed proteins that are exclusively shared by both fungi and focused on those proteins, which are overrepresented in certain functional categories, whereas proteins that have a functional annotation but are not enriched in functional classes are not listed (Table 3A). According to our expectation, *U. maydis *and *S. cerevisiae *have proteins in common that are essential for their uni-cellular life style. This includes the detoxification machineries and proteins that are involved in spore formation (Table 3; numbers represent the number of proteins in the cellular process listed in the table). In addition, both organisms contain 96 proteins that do not fall in any functional category and are therefore classified as "unknown function" The second set consists of proteins found in humans and *U. maydis *and also represents numerous cellular processes, including amino acid degradation, oxidation of fatty acids, mRNA splicing and modification, protein modification and degradation and G-protein mediated signalling (Table 3B). However, about one third of all proteins in this set (222; Table 3) are neither found in the FunCat nor in Clusters of eukaryotic orthologous groups in the COGs database [40]; [41], indicating that their function is not yet known ("unknown function"). Finally, we analyzed the *S. cerevisiae*-to-*H. sapiens *homology set and again found some cellular processes, including tRNA modification, secondary biosynthesis and protein fate and modification that are overrepresented in this protein set (Table 3C). In contrast to the previous comparison, only 46 of the 312 proteins that are unique for yeast and human were without functional prediction in FunCat or the COGs database.

**Table 3:** Functional classification of predicted proteins

	**Protein number**^¶^	**P-value**
A. U. maydis – S. cerevisiae*		
1. Biosynthesis*	75	1,7e-33
2. Spore formation/budding/cell polarity	68	1.9e-15
3. Transport**	49	1.3e-18
4. Ion homeostasis	36	4.2e-12
5. Stress response^$^	35	7.5e-11
6. Detoxification/Resistance	29	1.4e-10
7. Unknown function^†^	96	1
B. U. maydis – H. sapiens*		
1. mRNA splicing	38	6.9e-17
2. Protein modification/degradation	29	2.4e-04
3. G-protein-mediated signalling	17	3,6e-06
4. Amino acid degradation	14	4.4e-09
5. Oxidation of fatty acids	10	1.1e-05
6. Unknown function^†^	222	1
C. S. cerevisiae – H. sapiens*		
1. Biosynthesis, secondary^§^	105	3.0e-05
2. Protein fate, – modification	89	7.4e-06
3. Oxidative stress response	8	3.7e-03
4. tRNA modification	7	3.4e-03
5. Unknown function^†^	46	1

*Note that proteins can appear in more than one functional category. In addition, numerous proteins are assigned to additional functional classes that are not listed.

^¶ ^Only those predicted proteins were considered that (1) show more than 20% identity with either in both organisms and (2) show at least 10 % less homology in the third partner

*Amino acids/vitamins/cofactors

**Amino acids/lipids/carbohydrates

^$^Salt and oxidative stress

^§^Polyamines, vitamins, co-factors and other secondary products

^†^Predicted proteins are not found in the COGs or FunCat database

**Figure 5:**
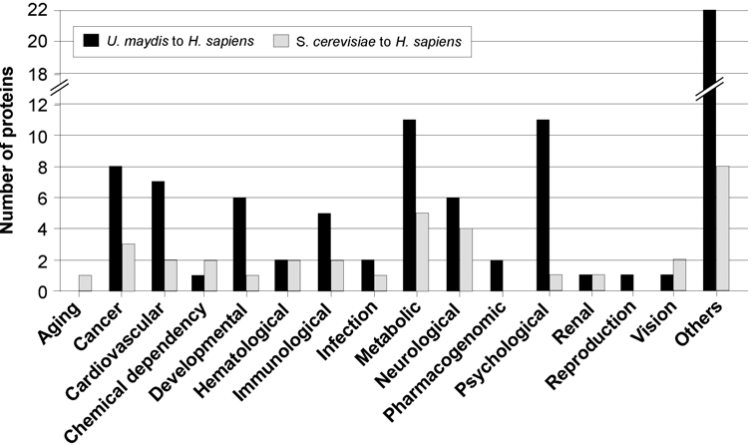
Proteins of the *U. maydis – H. sapiens *and *S. cerevisiae – H. sapiens *homology sets, which human counterpart have a predicted role in diseases. The classification was done according to main disease class in the genetic association database [47]. Note that *U. maydis *contains numerous genes that are thought to be involved in psychological disorders, such as ERCC1 and ERCC4.

Our analysis revealed that *U. maydis *and human share numerous proteins, and analysis of these might give insights into the molecular basis of human diseases. A good example for such a role for *U. maydis *is the analysis of the breast cancer susceptibility gene BRCA2 that confers a high risk of breast cancer and is the focus of cancer research since its discovery in the mid 1990s [42,43]. The activity of BRCA2 is not well-understood, which might in part be due to the fact that it was not found in the model system *S. cerevisiae*. Recently a BRCA2 homologue (Brh2; um03200) was identified in a screen for DNA-repair defective mutants [44], and it was shown that Brh2 enables recombinational DNA repair by controlling Rad51 [45,46]. Indeed, the *U. maydis *Rad51 is one of seven proteins that are implied in DNA repair and that are part of the 681 proteins identified to be highly conserved in humans and *U. maydis*. In order to gain insights into a role of *U. maydis *genes in human diseases we used the Genetic Association Database [47], which is an archive of all published knowledge of molecular disorders in humans. After adjusting the nomenclature (see Material and Methods) all proteins that matched were analyzed and then classified according to main disease classes. This analysis revealed that 42 proteins of the *U. maydis*-to-*H. sapiens *set are implicated in diseases, including cancer (8 proteins), cardiovascular disorders (7 proteins) and defects in metabolism (11). In addition, 22 proteins (Figure 5, "Others") were found that are implicated in various defined diseases or whose roles are not yet understood (Figure 5). Among these proteins are prominent oncogenes such as ERCC1 (um06219) and ERCC4 (um10396), which participate in excision repair of DNA, and when mutated cause various types of cancer, including non-small-cell lung cancer [48]. Another example is the KRAS protein (um01643), the GTP/GTP-binding protein acting in intracellular signal transduction (overview in [49]) that also is involved in cancer formation. Most interesting, we found several disease-related proteins that most likely are not present in the model system *S. cerevisiae *(≤ 14% identity; Table 4). This group includes ACADM (um01049) and ACADS (um01466), which encode acyl-CoA dehydrogenases (Table 4). Mutants in these genes cause medium-chain-acyl-CoA dehydrogenase deficiency [50] and short-chain-acyl-CoA dehydrogenase deficiency in humans [51].

**Table 4:** Disease-related proteins with counterparts in *U. maydis*

**Protein**	**Function**	**MIPS ID**^¶^	**Diseases**	U. maydis*	S. cerevisiae*	Ref.**
ACADM	acyl-CoA dehydrogenase	Um01049	acyl-CoA dehydrogenase deficiency	58.0	11.6	[68]
ACADS	acyl-CoA dehydrogenase	Um04833	acyl-CoA dehydrogenase deficiency	35.2	11.2	[69]
BZRP/PBR	Benzodiazepine receptor	Um06406	Cancer	27.4	10.1	[70]
CES1	Carboxylesterase	Um06071	Cancer	27.6	9.3	[71]
CLPTM1	unknown	Um02586	Cleft lip development	30.5	11.1	[72]
DDC	Dopa decarboxylase	Um06083	Parkinsons	34.7	11.0	[73]
DOCK3	Dedicator of cytokinesis 3	Um01178	Attention deficit hyperactivity disorder	23.1	12.3	[74]
EPHX1	Epoxide hydrolase	Um01938	Cancer	36.6	10.1	[74]
GCDH	glutaryl-CoA dehydrogenase	Um01335	glutaryl-CoA dehydrogenase deficiency	54.1	13.9	[75]
HEXA	Hexosaminidase A	Um00695	Tay-Sachs disease	20.1	12.7	[76]
HMGCL	Methylglutaryl CoA lyase	Um02001	methylglutaric CoA lyase deficiency	43.6	11.4	[77]
PIMT/PCMT1	Methyl-transferase	Um11483	Type 1 diabetes	48.9	9.3	[78]
PRSS16	serine protease	Um10091	Diabetes mellitus/susceptibility to autoimmunity	20.7	6.6	[79]
UGCG	Glucosyl-ceramide synthase	Um04496	induced apoptosis	22.3	8.1	[80]

^¶ ^Identification tag for search at the MIPS server [13]

* Values are given as percent identity to human protein, all the *U. maydis *hits are best bidirectional

** Reference for the human protein/disease

Finally, it is important to note that the yeast *S. cerevisiae *also shares 13 disease-related proteins with humans that are significantly less conserved in *U. maydis *(Figure 5), and 4 of these proteins are not present in *U. maydis *(< 10%). Interestingly, a third of all disease-related proteins in yeast are involved in diabetes. Among these is ADIPOR2 (YOL002c), an adiponectin receptor, which when deleted in mice promotes type 2 diabetes [52] and PYGL (YPR160c), which when mutated causes Glycogen phosphorylase deficiency, resulting in diabetes mellitus type 1 in humans [53].

## Conclusion

Fungal model systems, such as *S. cerevisiae *have greatly enhanced our knowledge of basic cell biology, which is in part due to numerous technical advantages and the published genome. However, some cellular processes that are important in humans are highly modified or are not even present in this fungus. Examples are microtubule-based transport that is essential in elongated neurons, but virtually absent from *S. cerevisiae*. Consequently, some proteins, such as the motor proteins kinesin-1 and kinesin-3 are not encoded by the genome of the budding yeast. Experimental evidence from work in *U. maydis *indicated that this fungus could fill in this gap. This organism has a long history as a model system for DNA repair and recombination, and shows additional similarities to human cells, such as long-distance transport and an open mitosis. Consequently, proteins like kinesin-1 and kinesin-3 are present and are highly conserved in *U. maydis*. Indeed, as much as ~10% of all *U. maydis *proteins have highly conserved counterparts in humans, but are not found or are significant less conserved in yeast. On the first glimpse this finding is surprising. However, genomic data indicate that fungi are an extremely divers taxon that covers around one billion years of divergent evolution [54]. The unexpectedly high conservation between *U. maydis *and humans might suggest that both organisms share some conserved cellular processes. However, it is important to note that functional predictions based on sequence homology can just be a first step towards an understanding of the cellular function. Careful experimental work is needed to further prove that *U. maydis *helps understanding the molecular basis of human diseases.

## Methods

### General Hardware and software

All software was compiled and run on one or more workstations with Fedora Linux or Alpha processor and Tru64 (formerly known as DEC-Unix) with compiled software for OSF v5.1. Unless otherwise noted, data mining and analyses was carried out using the Protein Mapping and comparison Tool PROMPT [55] an java application including the statistic package R, scripts written in Perl [56] or using own Java applications [57] developed with the open source platform Eclipse [58], each of which are available on request. In all cases, data is stored in mySQL 4/5 [59] or Oracle 9i [60] databases referred to as the MIPS Fungal Genome Databases (*U. maydis, S. cerevisiae*) and the Functional Catalogue Database (FunCatDB) [39].

### Homology analysis

In order to determine the homology set of the proteomes of *U. maydis*, *S. cerevisiae *and *H. sapiens *we used the SIMAP [61], an exhaustive application containing all significant pre-calculated similarity scores of the Smith-Waterman alignment algorithm [62] of protein pairs. The database contains more than 30 million proteins, including the current versions of the *S. cerevisiae *and *U. maydis *proteomes and all available human protein sequences and is well suited to speed up the search for biological meaningful hits [30]. In order to access this database, a java application was generated using the eclipse framework (version 3.2.1) for communication with the SIMAP retrieval layer (due to the huge size of the binary hit file 700 G and ongoing internal changes, a direct access is not permitted). This allowed for the access of further stored information (sequence ID, Smith-Waterman score, Identity score, Similarity score, overlap size of the pair-wise alignment and Start and Stop coordinates of the alignment in both proteins), as well as sorting and filtering to specific criteria.

Additionally, we implemented in our client application the "identity over length" sorting procedure to tackle the length dependency, an important task for transferring functional attributes. With help of this application and specifications, represented in single task files, a genome wide retrieving, sorting and taxonomic as well as homology filtering was feasible. Homologous proteins were identified using the whole-genome protein sets of *U. maydis *(MUMDB), e.g. *S. cerevisiae *(CYGD) and *H. sapiens *(UniProtKB Refseq). For various cut-offs of homology assignments for proteins and domains see also the specific result parts. Further we integrated a SIMAP access for data visualization of various taxonomy spaces (e.g. human) in each of our organism specific databases in the Genome Research Environment [63].

In contrast to our so far used "% identity over length" criteria we sorted the hits in the domain search by the standard e-value and considered the % identity in the overlap at a cut-off length of 50 aa.

### Sequence similarity background of structural (coiled-coil) domains

In order to predict coiled-coil domains we used Paircoil2 [64,65]). This tool uses pair-wise residue probabilities to detect coiled-coil motifs in protein sequence data and achieves 98% sensitivity and 97% specificity on known coiled coils in leave-family-out cross-validation. We took from each of the coiled-coil families (dynein, heat-shock factor, intermediate filament-like, kinesin-like, myosin, snare and tropomyosin) one representative *U. maydis *protein and detected the domain position (Table 2). Next we calculated the % identity over the entire protein for the 7 examples to each other and additionally to the *U. maydis *proteome. We filtered out the known related protein and thus we expected that like in yeast the coilome is around 20 % of the proteome [34]. Then we estimated the random % identity, which is by far lower than the range of the 30^th ^hit until the best unrelated hit shown in Table 2. Please note that 19.0% – 29.1% identity in the overlap region can be found for unrelated coiled-coil domain containing proteins.

### Functional data and analysis

The systematic classification of protein function is of great importance in functional genomics, as it organizes our thinking about the biological roles of proteins. The MIPS Functional Catalogue Database resource (FunCatDB; [39]) contains functional proteome information (collected literature as well as homology transformations) across organisms based on the hierarchical classification scheme FunCat [31]. Included in this database are high value manual functional annotation for the *S. cerevisiae *proteome as well as homology assignments to already manually annotated proteins, such as a quality value of > 25 % identity over length [35] for the *U. maydis *proteome. For each set of proteins we obtained and analyzed their functional distribution and the statistical significance of functional similarity groups, based on the p- value calculation of each group from the set against the corresponding whole genome reference set. To not miss a function due to possible incomplete annotation, we calculated with help of our automated Pedant analysis system [66,67] for all the *U. maydis *proteins the similarity to the Clusters of eukaryotic groups (KOGs; [40,41] using a cut-off of 20 % identity. The eukaryotic orthologous groups (KOGs) include proteins from 7 eukaryotic genomes including *H. sapiens *and *S. cerevisiae*. The current KOG set consists of 4852 clusters of orthologous proteins, which are annotated to a set of distinct main functions.

### Diseases and disorder analysis

In order to study the connection of homologous proteins to human diseases and disorders, we first mapped all human codes from SwissProt, ensemble, NCBI and Unicode to the official human gene nomenclature. We then applied the batch tool at the Genetic Association Database, an archive of human genetic association studies of complex diseases and disorders, for analysis of genetic association [47] and their classification in main disease classes. Additionally, we performed a literature search for all identified homologous human proteins to obtain the current and precise cellular functions as well as diseases.

## Authors' contributions

MM carried out the conception and design of the study and implemented the computational studies and statistical analysis. Further MM participated in the interpretation of data and the manuscript preparation. GS conceived of the study and participated in the interpretation of data and the manuscript preparation. All authors read and approved the final manuscript.

## Supplementary Material

Additional file 1 **List of all *Ustilago maydis *proteins with higher conservation to *Homo sapiens *than to *Saccharomyces cerevisiae***. Listed are the *U. maydis *code, the %identity over protein length to *H. sapiens *and the % identity over protein length to *S. cerevisiae*, based on the Smith-Waterman comparison of all proteins between the genomes, a flag (1) for a best bidirectional hit, *H. sapiens *code, the alternative best reciprocal *U. maydis *code, the %identity over protein length of the best reciprocal hit and comments (The *U. maydis *annotation is still ongoing, please find here existing new gene models). **List of alternative Best Recipropal Hits of the *U. maydis*-to-*H. sapiens *set**. Listed are all best recipropal *U. maydis *codes, their amount and their relation to the *U. maydis*-to-*H. sapiens *set. Click here for file
