# Supplementary material for: The fungus Ustilago maydis and humans share disease-related proteins that are not found in Saccharomyces cerevisiae
Source: BMC Genomics. 2007 Dec 20;8:473. doi: 10.1186/1471-2164-8-473 (PMC2262911; doi:10.1186/1471-2164-8-473)
Supplement: Additional file 1 — List of all Ustilago maydis proteins with higher conservation to Homo sapiens than to Saccharomyces cerevisiae. Listed are the U. maydis code, the %identity over protein length to H. sapiens and the % identity over protein length to S. cerevisiae, based on the Smith-Waterman comparison of all proteins between the genomes, a flag (1) for a best bidirectional hit, H. sapiens code, the alternative best reciprocal U. maydis code, the %identity over protein length of the best reciprocal hit and comments (The U. maydis annotation is still ongoing, please find here existing new gene models). List of alternative Best Recipropal Hits of the U. maydis-to-H. sapiens set. Listed are all best recipropal U. maydis codes, their amount and their relation to the U. maydis-to-H. sapiens set. [file 1471-2164-8-473-S1.pdf]

List of all *Ustilago maydis* proteins with higher conservation to *Homo sapiens* than to *Saccharomyces cerevisiae* . Listed are the *U. maydis* code, the %identity over protein length to *H. sapiens* and the % identity over protein length to *S. cerevisiae* , based on the Smith-Waterman comparison of all proteins between the genomes, a flag (1) for a best bidirectional hit, *H. sapiens* code, the alternative best reciprocal *U. maydis* code, the %identity over protein length of the best reciprocal hit and comments (The U. maydis annotation is still ongoing, please find here changed gene models). **List of alternative Best Rezipropal Hits of the *U.maydis* -to-*H.sapiens* set.** Listed are all best recipropal *U. maydis* codes, their amount and their relation to the *U.maydis* -to-*H.sapiens* set. BBH=BestBidirectional Hit BRH=BestReciprocal Hit

U.maydis to-_H.sapiens_ homologous proteins from full protein analysis

U.maydis code	%identity to *H.sapiens*	%identity to *S.cerevisiae*	BBH	*H.sapiens* code	BRH *U.maydis* code	% identity to BRH	comments
um00008	25,0	9,3	1	NP_689629			
um00017	22,9	11,1	0	ICMT_HUMAN	um11682	32,8	BRH not in *U.maydis* -to-*H.sapiens* set
um00025	21,3	9,6	1	NAP1_HUMAN			
um00029	39,8	16,6	1	MIOX_HUMAN			
um00030	21,3	11	1	PROD2_HUMAN			
um00037	33,5	16,9	1	NP_001018070			
um00040	25,8	9,9	1	DNJC8_HUMAN			
um00076	20,5	7,9	1	NP_689898			
um00118	56,9	14	1	UGDH_HUMAN			
um00131	35,3	24,6	1	QORX_HUMAN			
um00152	22,4	11,1	1	A2RRM2_HUMAN			
um00154	22,5	10,5	0	EST3_HUMAN	um06071	26,1	BRH is member of the *U.maydis* -to-*H.sapiens* set
um00172	21,5	8,0	1	REC8_HUMAN			
um00182	30,1	8,7	1	Q504V3_HUMAN			
um00199	23,6	10,2	1	Q53HR0_HUMAN			
um00219	22,5	7,6	1	NP_060306			
um00305	30,0	7,3	1	SF3A1_HUMAN			
um00347	29,4	5,3	1	TBCD_HUMAN			
um00353	24,9	10,2	1	JMJD4_HUMAN			
um00362	20,9	8,8	0	MOSC1_HUMAN	um00984	23,7	BRH is member of the *U.maydis* -to-*H.sapiens* set
um00364	37,6	21,4	1	PRP31_HUMAN	um00364.2	35,2	*U. maydis* gene model changed
um00379	32,0	9,0	1	IF36_HUMAN			
um00381	39,2	14,3	1	NDUA9_HUMAN			
um00388	20,5	7,8	0	XP_001131108	um05820	21,0	BRH not in *U.maydis* -to-*H.sapiens* set
um00423	24,6	12,8	1	Q53GC7_HUMAN			
um00424	31,3	11,8	0	CHDH_HUMAN	um01872	32,1	BRH is member of the *U.maydis* -to-*H.sapiens* set
um00460	24,3	10,3	1	EXOS7_HUMAN			
um00512	33,5	10,3	1	NDUS4_HUMAN			
um00522	21,5	9,8	1	NP_733837			
um00523	24,0	12,5	1	CNOT2_HUMAN			
um00560	61,9	46,5	1	Q6FI27_HUMAN			
um00584	60,1	45,0	1	Q53EZ1_HUMAN			
um00590	21,0	10,2	1	NP_056200			
um00595	59,6	41,4	1	AATM_HUMAN			
um00599	23,3	8,8	1	DPOLM_HUMAN			

um00627	20,9	4,9	1	A6NEH8_HUMAN			
um00634	52,1	10,4	1	Q6IPW4_HUMAN			
um00639	20,2	5,5	1	NP_056046			
um00654	54,8	39,8	1	Q53YD8_HUMAN			
um00655	23,0	8,7	1	Q8N909_HUMAN			
um00660	46,3	33,0	1	AP1G1_HUMAN	um00660.2	45,2	*U. maydis* gene model changed
um00666	25,1	8,8	1	THEX1_HUMAN			
um00667	31,4	17,6	1	RBM22_HUMAN			
um00673	26,9	7,1	1	PKHA8_HUMAN			
um00694	23,5	9,5	0	ACADS_HUMAN	um01049	38,7	BRH is member of the *U.maydis*-to-*H.sapiens* set
um00695	20,1	9,0	1	HEXB_HUMAN			HEXA (*H.sapiens* gene name)
um00699	22,3	7,8	1	DGC14_HUMAN			
um00703	67,1	35,2	1	GBB1_HUMAN			
um00711	26,9	7,3	1	PSME3_HUMAN			
um00719	23,3	8,6	1	Q6ZVY3_HUMAN			
um00734	20,2	6,9	0	XP_001128001	um01179	21,9	BRH not in *U.maydis*-to-*H.sapiens* set
um00737	53,1	25,8	1	SF3B3_HUMAN			
um00748	59,3	4,9	1	SF3B5_HUMAN			
um00749	20,1	6,0	1	Q86VM2_HUMAN			
um00752	20,2	5,2	1	Q96PG8_HUMAN			
um00774	80,3	66,8	1	RAC1_HUMAN			
um00775	21,1	9,4	1	UBE2O_HUMAN			
um00817	51,3	17,5	1	CSN2_HUMAN			
um00878	20,3	9,1	1	NP_006687			
um00928	23,7	5,8	1	CA074_HUMAN			
um00950	27,1	10,7	1	Q5T8L2_HUMAN			
um00984	24,7	9,2	1	NP_073583			
um01005	55,0	7,7	1	Q4LE36_HUMAN			
um01011	34,8	21,9	1	DBR1_HUMAN			
um01031	37,3	14,1	1	NAT13_HUMAN			
um01041	24,7	4,9	1	XP_001132867			
um01045	24,0	12,1	1	GPTC1_HUMAN			
um01049	58,0	9,0	1	ACADM_HUMAN			ACADM (*H.sapiens* gene name)
um01058	42,9	30,2	1	AP3S2_HUMAN			
um01087	46,3	33,2	1	CUL1_HUMAN			
um01099	45,9	13,1	1	HCDH_HUMAN			
um01166	21,7	9,4	1	NP_060506			
um01174	36,9	26,7	1	DDX46_HUMAN			
um01178	23,2	7,9	1	DOCK3_HUMAN			DOCK3 (*H.sapiens* gene name)
um01195	26,4	8,5	1	SQRD_HUMAN			
um01198	22,0	11,0	1	DEOC_HUMAN			
um01200	22,6	10,7	0	MYST2_HUMAN	um05240	26,6	BRH not in *U.maydis*-to-*H.sapiens* set
um01207	29,3	18,7	1	DJB12_HUMAN			
um01214	22,4	4,9	1	G7C_HUMAN			
um01219	23,1	8,5	1	XP_001133255			
um01228	35,5	8,4	1	IF36I_HUMAN			

um01242	40,2	28,5	1	A6NIB3_HUMAN			
um01266	27,5	9,0	1	CTBL1_HUMAN			
um01311	37,8	4,9	1	NP_057049			
um01321	42,8	7,4	1	IF37_HUMAN			
um01323	35,3	21,7	1	ISY1_HUMAN			
um01330	20,8	4,9	1	XP_933002			
um01357	25,5	6,6	1	Q6GMV3_HUMAN			
um01367	44,2	26,8	1	Q53ET1_HUMAN			
um01378	21,6	6,7	1	RGAG1_HUMAN			
um01425	52,8	10,3	1	NP_000178			
um01433	39,7	10,8	1	Q86YB7_HUMAN			
um01436	20,7	7,6	1	A5YW33_HUMAN			
um01466	21,4	8,3	0	ACADS_HUMAN	um01049	38,7	BRH is member of the *U.maydis*-to-*H.sapiens* set
um01512	29,6	16,6	1	NP_064505			
um01518	21,0	9,5	1	O60382_HUMAN			
um01533	34,7	13,9	1	SAT2_HUMAN			
um01553	23,8	6,8	1	Q6UWP3_HUMAN			
um01561	41,6	28,4	1	NP_057030			
um01578	34,8	14,6	1	NP_067054			
um01607	27,4	8,3	1	TSN_HUMAN			
um01619	41,3	28,5	1	GPDM_HUMAN			
um01643	56,2	34,3	1	NP_004976			
um01651	75,3	57,4	1	A5YM50_HUMAN			
um01677	23,5	7,6	0	XP_001127031	um15060	21,5	BRH not in *U.maydis*-to-*H.sapiens* set
um01711	29,5	7,4	1	CHDH_HUMAN			
um01718	56,3	18,7	1	Q8TDQ7_HUMAN			
um01735	54,5	33,8	1	RAB4B_HUMAN			
um01747	24,8	9,5	1	Q59H27_HUMAN			
um01777	28,5	10,1	1	NP_004861			
um01794	27,5	11,8	1	CECR1_HUMAN			
um01797	21,9	11,6	0	NPAL1_HUMAN	um02993	28,0	BRH is member of the *U.maydis*-to-*H.sapiens* set
um01800	24,8	9,4	1	MOS2L_HUMAN			
um01815	36,7	19,6	1	CBR1_HUMAN			
um01850	21,8	9,5	0	NP_001007101	um11983	33,6	BRH is member of the *U.maydis*-to-*H.sapiens* set
um01852	27,5	8,1	0	HYEP_HUMAN	um01938	30,6	BRH not in *U.maydis*-to-*H.sapiens* set
um01858	26,0	10,8	1	SPEB_HUMAN			
um01872	32,1	10,3	1	CHDH_HUMAN			
um01923	43,0	10,2	1	CG010_HUMAN			
um01935	39,8	5,2	1	Q5VYU8_HUMAN			
um01937	23,0	12,2	1	ASM_HUMAN			
um01942	31,7	13,3	1	DPEP1_HUMAN			
um01974	34,8	7,7	1	Q59FF0_HUMAN			
um01986	40,5	23,1	1	Q5VVZ1_HUMAN			
um01992	30,1	11,2	1	AC1L2_HUMAN			
um02001	43,6	13,1	1	HMGCL_HUMAN			HMGCL (*H.sapiens* gene name)
um02016	39,7	26,0	1	Q6IB11_HUMAN			

um02075	21,9	8,6	1	CAN7_HUMAN			
um02079	49,1	36,9	1	RS10_HUMAN			
um02081	53,9	33,0	1	NP_001672			
um02097	27,6	17,3	0	Q5VYU7_HUMAN	um01935	38,3	BRH is member of the *U.maydis*-to-*H.sapiens* set
um02105	37,6	11,1	0	HCDH_HUMAN	um01099	48,9	BRH is member of the *U.maydis*-to-*H.sapiens* set
um02109	29,8	6,9	0	Q6UJX3_HUMAN	um11470	31,4	BRH not in *U.maydis*-to-*H.sapiens* set
um02115	47,5	8,0	1	Q7L5Y1_HUMAN			
um02125	31,3	9,9	1	DCE2_HUMAN			
um02144	30,3	11,7	1	CRY2_HUMAN			
um02185	60,2	34,1	1	PPIL3_HUMAN			
um02189	36,9	16,3	1	Q546Z2_HUMAN			
um02208	37,8	26,0	1	Q7Z3W5_HUMAN			
um02211	26,2	11,2	1	Q9BUL2_HUMAN			
um02281	43,8	29,7	1	PRP6_HUMAN			
um02305	24,6	11,2	1	TTC9C_HUMAN			
um02366	20,7	4,9	1	SRP09_HUMAN			
um02378	31,7	21,6	1	NP_004913			
um02384	23,5	4,9	1	CS056_HUMAN			
um02401	30,8	20,4	1	PABP2_HUMAN			
um02405	24,4	14,0	1	A6NIG1_HUMAN			
um02412	34,9	18,7	0	Q53XX5_HUMAN	um10149	38,9	BRH is member of the *U.maydis*-to-*H.sapiens* set
um02415	43,1	33,0	1	ASF1A_HUMAN			
um02425	51,2	23,3	1	Q7Z530_HUMAN			
um02426	63,0	43,4	1	UB2G1_HUMAN			
um02437	35,1	7,5	1	NDUA6_HUMAN			
um02469	25,5	13,6	1	SRR35_HUMAN			
um02471	21,6	5,9	1	XPO4_HUMAN			
um02478	28,5	5,4	1	NP_071401			
um02485	61,0	50,9	1	RAB5C_HUMAN			
um02517	48,9	30,1	0	GNAI1_HUMAN	um10177	55,4	BRH is member of the *U.maydis*-to-*H.sapiens* set
um02530	25,1	4,9	1	Q5T626_HUMAN			
um02541	24,3	4,9	1	XP00131632			
um02586	30,4	8,9	1	CLPT1_HUMAN			CLPTM1 (*H.sapiens* gene name)
um02591	26,3	11,3	1	RAD21_HUMAN			
um02609	42,4	21,7	1	Q53TM1_HUMAN			
um02618	40,4	12,8	1	SNW1_HUMAN	um02618.2	41,5	*U. maydis* gene model changed
um02632	26,4	12,7	1	NUDC_HUMAN			
um02645	77,5	56,0	1	TXN4A_HUMAN			
um02651	30,6	9,9	1	Q6QHC5_HUMAN			
um02655	20,3	8,6	1	Q9NTB1_HUMAN			
um02667	51,0	10,6	1	SCOT_HUMAN			
um02676	44,8	33,6	1	Q5JY65_HUMAN			
um02693	29,2	8,2	1	NP_258428			
um02729	36,0	16,0	1	HM13_HUMAN			
um02740	29,3	16,6	1	LYAG_HUMAN			
um02743	37,8	4,9	1	Q7Z623_HUMAN			

um02777	33,4	20,8	1	A6NFL0_HUMAN			
um02794	34,2	23,1	1	PSMD9_HUMAN			
um02797	22,0	5,2	1	TTC9B_HUMAN			
um02824	29,6	9,2	1	Q6IN49_HUMAN			
um02864	21,5	8,9	0	CHLE_HUMAN	um11540	26,7	BRH is member of the *U.maydis* -to-*H.sapiens* set
um02868	54,5	35,8	1	Q6IBM8_HUMAN			
um02883	24,0	5,8	1	PFD1_HUMAN			
um02901	24,2	4,9	1	Q9NVE8_HUMAN			
um02912	26,1	15	1	NP_061897			
um02913	28,5	16,5	1	Q6MZT3_HUMAN			
um02925	20,5	7,7	0	Q01212_HUMAN	um01382	21,1	BRH not in *U.maydis* -to-*H.sapiens* set
um02944	24,6	11,0	1	NP_060668			
um02959	41,5	27,3	1	Q4VWZ6_HUMAN			
um02960	55,5	44,3	1	2AAA_HUMAN			
um02984	21,4	8,3	0	GCDH_HUMAN	um01335	54,1	BRH is member of the *U.maydis* -to-*H.sapiens* set
um02993	28,4	8,9	1	NIPA2_HUMAN			
um03015	26,5	12,3	1	CA128_HUMAN			
um03017	20,8	6,8	1	XP_938564			
um03020	25,0	7,7	1	THYN1_HUMAN			
um03074	52,4	39,2	1	BTF3_HUMAN			
um03089	21,4	9,3	1	GPKOW_HUMAN			
um03100	30,5	4,9	1	DLRB2_HUMAN			
um03134	23,3	10,3	1	CX056_HUMAN			
um03144	54,5	36,6	1	AP2M1_HUMAN			
um03147	35,4	23,3	1	Q6PK82_HUMAN			
um03158	24,5	10,1	0	Q9BVL8_HUMAN	um11556	25,3	BRH is member of the *U.maydis* -to-*H.sapiens* set
um03164	46,3	28,2	1	LIS1_HUMAN			
um03218	28,6	12,9	1	SCAM2_HUMAN			
um03220	20,5	10,0	0	ECHM_HUMAN	um11556	54,1	BRH is member of the *U.maydis* -to-*H.sapiens* set
um03227	38,4	28,2	1	A6NLW3_HUMAN			
um03237	69,7	55,7	1	RS20_HUMAN			
um03246	30,6	10,0	0	CHDH_HUMAN	um01872	32,1	BRH is member of the *U.maydis* -to-*H.sapiens* set
um03252	39,9	28,3	1	Q6IBN0_HUMAN			
um03260	21,7	8,5	1	Q5T6T8_HUMAN			
um03264	43,5	4,9	1	XDH_HUMAN			
um03277	23,0	7,2	1	Q96F88_HUMAN			
um03290	72,6	57,3	1	RAD51_HUMAN			
um03292	22,7	6,3	1	CN130_HUMAN			
um03298	60,3	41,4	1	THIM_HUMAN			
um03308	37,7	8,4	1	AMPL_HUMAN			
um03336	42,0	24,5	1	STRAP_HUMAN			
um03362	22,2	11,8	1	Q5QPM7_HUMAN			
um03372	23,6	6,6	1	SENP8_HUMAN			
um03409	40,1	8,8	0	FAAA_HUMAN	um11337.2	44,1	BRH is member of the *U.maydis* -to-*H.sapiens* set
um03442	42,7	22,0	1	Q53FW4_HUMAN			
um03443	44,1	32,9	1	UBE2W_HUMAN			

um03445	25,6	4,9	1	Q5SXR8_HUMAN			
um03456	22,4	10,2	1	XP_931330			
um03477	34,3	8,9	1	CAND1_HUMAN			
um03478	30,8	17,0	1	PCID2_HUMAN			
um03491	30,4	9,7	1	CSN7A_HUMAN			
um03530	29,5	4,9	1	TIM16_HUMAN			
um03551	27,7	13,6	0	CHDH_HUMAN	um01872	32,1	BRH is member of the *U.maydis*-to-*H.sapiens* set
um03562	20,7	4,9	1	XP_001129977			
um03580	35,6	9,7	1	Q5T0M6_HUMAN			
um03615	29,0	7,8	1	CHDH_HUMAN	um01872	32,1	BRH is member of the *U.maydis*-to-*H.sapiens* set
um03628	26,5	5,1	1	NP_060417			
um03642	20,4	5,2	1	Q5TCP6_HUMAN			
um03665	44,4	24,3	1	AL7A1_HUMAN			
um03701	21,0	10,9	1	MUC1_HUMAN			
um03717	20,7	9,4	1	NP_055754			
um03738	50,4	40,3	1	U520_HUMAN			
um03756	20,4	6,3	1	Q2TAK1_HUMAN			
um03759	44,6	20,9	1	CSN5_HUMAN			
um03784	29,3	16,3	1	NP_001077362			
um03803	70,7	37,6	1	TBG1_HUMAN			
um03817	25,0	7,6	1	XP_373106			
um03833	78,9	67,8	1	Q5U0I6_HUMAN			
um03842	36,2	17,8	1	XAB2_HUMAN			
um03883	21,1	8,6	1	WDFY3_HUMAN			
um03893	46,6	13,3	1	Q701P4_HUMAN			
um03910	91,3	61,1	1	CALM_HUMAN			
um03930	22,6	5,8	1	Q53FD2_HUMAN			
um03935	20,2	10,0	1	Q8N6Z6_HUMAN			
um03936	58,6	44,4	1	DHX8_HUMAN			
um03941	35,5	17,8	1	Q6UX04_HUMAN			
um03964	29,5	16,3	1	Q5VT54_HUMAN			
um03973	32,4	21,4	1	ST32A_HUMAN			
um03990	27,5	15,0	0	Q5STP7_HUMAN	um10898	30,7	BRH not in *U.maydis*-to-*H.sapiens* set
um04006	22,1	4,9	1	K0907_HUMAN			
um04009	30,5	18,7	1	CA069_HUMAN			
um04044	29,7	12,1	1	CHDH_HUMAN			
um04061	39,4	23,0	1	Q5T945_HUMAN			
um04072	27,1	5,5	1	Q9H875_HUMAN			
um04109	23,3	10,9	0	CP2S1_HUMAN	um03662	23,7	BRH not in *U.maydis*-to-*H.sapiens* set
um04149	27,2	15,0	1	RBM18_HUMAN			
um04165	26,5	4,9	1	RFA3_HUMAN			
um04183	29,1	5,2	1	SNUT3_HUMAN			
um04188	48,4	34,4	1	PRP16_HUMAN			
um04189	22,8	10,9	0	Q8IZB0_HUMAN	um00005	23,0	BRH is member of the *U.maydis*-to-*H.sapiens* set
um04194	32,9	6,9	1	FA32A_HUMAN			
um04210	21,1	9,4	1	NP_116098			

um04218	35,4	23	1	KIF5C_HUMAN			
um04219	30,3	8,2	1	NP_659486			
um04277	49,4	38,8	1	NAT5_HUMAN			
um04310	33,0	9,0	1	PPWD1_HUMAN			
um04312	26,5	9,6	1	ACTN3_HUMAN			
um04318	24,2	9,5	1	PCYOX_HUMAN			
um04346	24,3	12,6	1	SYMPK_HUMAN			
um04352	65,0	33,4	1	MOL1A_HUMAN			
um04362	25,0	9,2	1	CP8B1_HUMAN			
um04365	26,3	15,0	1	RCC2_HUMAN			
um04372	25,4	9,1	1	A6NC83_HUMAN			
um04376	21,1	10,9	1	ERMP1_HUMAN			
um04382	43,8	16,8	1	MCCA_HUMAN			
um04390	21,3	11,2	1	Q6PJB9_HUMAN			
um04397	47,9	31,1	1	NP_002261			
um04411	43,1	23,8	1	CDC5L_HUMAN			
um04426	31,0	10,6	1	SCPDH_HUMAN			
um04446	50,0	27,8	1	LSM4_HUMAN			
um04448	32,2	19,1	0	Q6IBG1_HUMAN	um03910	36,7	BRH is member of the *U.maydis*-to-*H.sapiens* set
um04463	28,9	16,7	1	LMA2L_HUMAN			
um04494	24,3	10,1	1	NP_078988			
um04496	22,3	6,8	1	CEGT_HUMAN			UGCG (*H363* gene name)
um04498	20,2	9,6	1	Q86YL6_HUMAN			
um04518	27,1	9,8	1	Q14CA3_HUMAN			
um04542	67,9	57,2	1	UBC9_HUMAN			
um04553	31,2	6,5	1	Q6FIA7_HUMAN			
um04587	51,9	27,9	1	DDX41_HUMAN			
um04598	35,0	19,2	1	DC1I2_HUMAN			
um04599	36,1	14,5	1	PR38A_HUMAN			
um04601	20,9	7,4	1	Q8N5S0_HUMAN			
um04611	61,5	47,1	1	SKP1_HUMAN			
um04654	39,2	26,0	1	Q53G21_HUMAN			
um04659	50,2	29,7	1	GLPK_HUMAN			
um04666	23,4	11,7	1	THEM2_HUMAN			
um04667	29,7	8,4	1	DAPAL_HUMAN			
um04673	32,9	15,7	1	RECQ1_HUMAN			
um04677	20,3	10,1	1	MUC1_HUMAN			
um04679	38,8	27,2	1	Q5SZ63_HUMAN			
um04722	30,9	20,3	1	RPB4_HUMAN			
um04723	38,3	25,1	1	WDR5_HUMAN			
um04736	40,7	29,4	1	CHMP6_HUMAN			
um04752	43,5	23,9	1	PRP17_HUMAN			
um04758	36,9	12,9	1	PPIL2_HUMAN			
um04770	23,9	8,1	1	SPF30_HUMAN			
um04781	66,7	53,1	1	SMD2_HUMAN			
um04785	29,1	17,6	1	HUS1_HUMAN			

um04799	22,3	10,4	1	WIBG_HUMAN			
um04812	20,5	8,8	1	A6NLF1_HUMAN			
um04833	53,3	10,8	1	Q53XZ9_HUMAN			ACADS / IVD (*H.sapiens* gene name)
um04852	21,6	10,3	1	NP_942592			
um04860	32,6	13,7	1	CI114_HUMAN			
um04900	29,2	17,5	1	HIRA_HUMAN			
um04918	21,5	9,3	1	Q5VU05_HUMAN			
um04951	34,9	12,9	1	NP_006416			
um04965	26,1	4,9	1	SRP14_HUMAN			
um04987	28,3	4,9	1	4EBP1_HUMAN			
um04994	24,9	14,1	1	NP_005207			
um04995	26,4	7,8	1	NKAP_HUMAN			
um05037	34,6	22,0	0	GRHPR_HUMAN	um04061	39,4	BRH is member of the *U.maydis*-to-*H.sapiens* set
um05061	22,5	7,3	1	L2HDH_HUMAN			
um05068	28,9	11,8	1	Q1HA39_HUMAN			
um05131	40,5	25,1	1	Q8NEW5_HUMAN			
um05141	25,6	12,8	1	POLK_HUMAN			
um05159	29,7	12,3	1	ZMAT2_HUMAN			
um05161	23,9	11,5	1	PHLP_HUMAN			
um05204	30,4	10,2	1	PHOP2_HUMAN			
um05229	22,6	4,9	1	MANBA_HUMAN			
um05238	21,2	4,9	1	A5X8Z8_HUMAN			
um05241	56,7	36,1	1	NP_036565			
um05247	41,7	10,9	0	MCCC2_HUMAN	um11932	52,9	BRH is member of the *U.maydis*-to-*H.sapiens* set
um05274	20,5	9,8	1	NP_001529			
um05275	35,1	22,8	1	PDK3_HUMAN			
um05282	49,1	34,5	1	CHMP5_HUMAN			
um05292	25,8	12,0	1	VPS18_HUMAN			
um05312	20,6	5,8	1	NMNA3_HUMAN			
um05335	26,8	10,1	1	CT043_HUMAN			
um05363	26,8	10,2	1	U2AF2_HUMAN			
um05390	39,1	15,7	1	PXMP4_HUMAN			
um05394	21,4	4,9	1	CU087_HUMAN			
um05415	33,0	18,6	1	DNJC7_HUMAN			
um05434	56,3	42,9	1	SBDS_HUMAN			
um05454	37,4	18,6	1	NP_006833			
um05503	24,6	13,4	1	VATH_HUMAN			
um05511	76,8	65,9	1	RAB7A_HUMAN			
um05512	23,8	7,2	1	Q86TG6_HUMAN			
um05547	26,6	15,6	1	AG10B_HUMAN			
um05563	38,0	20,7	1	A2A2W2_HUMAN			
um05572	44,2	21,4	1	TRP13_HUMAN			
um05573	22,3	6,2	1	Q6ZPC8_HUMAN			
um05580	78,0	64,9	1	ARF6_HUMAN			
um05592	30,7	4,9	1	A3KMH1_HUMAN			
um05598	21,6	8,0	1	NDUA8_HUMAN			

um05608	31,7	9,0	1	RU1C_HUMAN			
um05617	23,9	8,2	1	NP_001077			
um05625	28,7	14,0	1	Q96SA0_HUMAN			
um05641	26,6	12,2	1	Q6FGQ4_HUMAN			
um05665	38,9	18,1	1	Q6PIN5_HUMAN			
um05676	26,9	14,7	1	Q96QU6_HUMAN			
um05677	29,1	15,0	1	CLYBL_HUMAN			
um05678	29,8	10,2	1	GSTK1_HUMAN			
um05703	28,3	15,0	1	OXDA_HUMAN			
um05708	24,6	9,5	1	CF015_HUMAN			
um05719	22,2	7,0	1	A6NFJ2_HUMAN			
um05726	32,1	18,6	1	Q5W0K5_HUMAN			
um05748	38,7	4,9	1	NECP2_HUMAN			
um05754	52,9	41,4	1	Q53XN3_HUMAN			
um05773	21,5	10,5	1	Q86X51_HUMAN			
um05828	81,1	70,7	1	TBB2B_HUMAN			
um05829	65,8	4,9	1	MGN2_HUMAN			
um05834	34,1	17,4	1	A6NCK0_HUMAN			
um05856	22,8	12,2	1	XP_001127665			
um05938	41,1	11,1	1	PIR_HUMAN			
um05961	39,4	9,2	1	Q3KT79_HUMAN			
um05964	26,5	14,9	0	NP_001020366	um06071	28,0	BRH is member of the *U.maydis*-to-*H.sapiens* set
um06009	36,3	23,4	1	A4D1D2_HUMAN			
um06029	29,4	15,2	1	Q8TCM6_HUMAN			
um06033	28,0	4,9	1	MACD2_HUMAN			
um06036	25,0	7,9	1	CCD12_HUMAN			
um06071	27,6	8,2	1	EST1_HUMAN			CES1 (*H.sapiens* gene name)
um06082	33,6	12,9	1	NIPS2_HUMAN			
um06083	35,1	8,6	1	Q53Y41_HUMAN			DDC (*H.sapiens* gene name)
um06093	29,7	7,0	1	S35A3_HUMAN			
um06118	31,6	12,6	1	TPP1_HUMAN			
um06129	74,9	62,2	1	Q6IBQ2_HUMAN			
um06135	31,3	4,9	1	TMM19_HUMAN			
um06140	34,5	15,8	1	PEF1_HUMAN			
um06151	26,7	12,8	1	Q5VT41_HUMAN			
um06180	20,9	4,9	1	Q96GF5_HUMAN			
um06185	43,6	11,0	1	Q5SQN6_HUMAN			
um06191	22,3	9,2	1	Q5M7Z5_HUMAN			
um06210	22,3	8,6	1	ARS2_HUMAN			
um06219	34,1	17,9	1	ERCC1_HUMAN			
um06222	24,5	8,0	1	XP_001125906			
um06234	46,9	36,0	1	PSA6_HUMAN			
um06251	35,5	12,0	1	KIF1A_HUMAN			
um06265	20,3	6,7	1	O00370_HUMAN			
um06292	58,9	38,3	1	VP26B_HUMAN			
um06302	22,8	5,2	1	XP_001129289			

um06326	30,3	18,2	1	FAH2A_HUMAN			
um06343	21,4	4,9	1	Q96FE3_HUMAN			
um06387	31,9	21,2	1	TAF9_HUMAN			
um06400	43,1	13,8	1	ACD10_HUMAN			
um06406	27,4	5,8	1	TSPO_HUMAN			BZRP/PBR (*H.sapiens* gene name)
um06422	31,2	8,5	1	ACD10_HUMAN			
um06477	23,5	10,3	1	CI078_HUMAN			
um10019	33,1	6,4	1	MOS1B_HUMAN			
um10022	26,7	11,4	1	MMTA2_HUMAN			
um10032	32,3	5,3	1	NAT9_HUMAN			
um10042	26,1	12,3	1	NP_060288			
um10045	24,2	4,9	1	XP_950487			
um10050	59,2	37,9	1	LSM5_HUMAN			
um10062	22,6	9,6	1	Q53XR0_HUMAN			
um10068	24,3	11,4	1	Q6ZNF0_HUMAN			
um10075	55,1	41,1	1	URM1_HUMAN			
um10077	47,1	19,8	1	Q8IWW8_HUMAN			
um10081	25,4	5,9	1	HDHD2_HUMAN			
um10082	38,4	15,9	1	DCTN5_HUMAN			
um10088	35,5	21,2	0	Q8NEW5_HUMAN	um05131	40,4	BRH is member of the *U.maydis*-to-*H.sapiens* set
um10091	20,7	8,1	1	TSSP_HUMAN			PRSS16 (*H.sapiens* gene name)
um10097	36,9	11,4	1	CT011_HUMAN			
um10106	28,3	15,4	1	A6NCP3_HUMAN			
um10112	20,9	9,5	1	CC137_HUMAN			
um10118	52,9	34,6	1	CCD25_HUMAN			
um10133	28,5	17,5	1	A5LGM8_HUMAN			
um10142	50,2	9,9	1	CPSF5_HUMAN			
um10144	28,0	5,6	1	SUOX_HUMAN			
um10146	70,7	52,9	1	Q5VLR4_HUMAN			
um10149	38,9	18,7	1	RBM3_HUMAN			
um10160	23,9	12,4	1	K0090_HUMAN			
um10162	26,9	16,6	1	HNRPG_HUMAN			
um10164	38,5	24,4	1	UBE2S_HUMAN			
um10177	56,3	38,4	1	GNAI2_HUMAN			
um10214	22,3	9,4	1	CRTC1_HUMAN			
um10235	74,9	64,6	1	PP4C_HUMAN			
um10239	35,7	22,1	1	Q549U1_HUMAN			
um10240	54,6	10,3	0	GCDH_HUMAN	um01335	54,1	*U. maydis* gene model changed
um10253	41,2	26,3	1	NP_055958	um10253.2	39,2	*U. maydis* gene model changed
um10267	49,2	4,9	1	SERF2_HUMAN			
um10273	34,2	19,4	1	ECH1_HUMAN			
um10297	31,5	11,1	1	PRP18_HUMAN			
um10316	23,1	8,2	0	VPS39_HUMAN	um15000.2	11,3	*U. maydis* gene model changed
um10326	20,1	8,9	1	RPKL1_HUMAN	um10326.2	12,7	*U. maydis* gene model changed
um10342	22,2	7,5	0	Q86X51_HUMAN	um05773	23,2	BRH is member of the *U.maydis*-to-*H.sapiens* set
um10344	21,9	6,6	1	Q2VPA5_HUMAN			

um10346	28,0	15,8	1	Q6PJM8_HUMAN			
um10376	37,4	23,6	1	CI041_HUMAN			
um10381	66,4	40,4	1	SMD1_HUMAN			
um10396	38,4	28,1	1	ERCC4_HUMAN	um10396.2	38,1	*U. maydis* gene model changed
um10399	24,1	14,0	1	NP_001092872			
um10403	20,1	4,9	1	Q9ULY9_HUMAN			
um10418	22,3	4,9	1	LV601_HUMAN			
um10420	24,5	10,8	1	SPO11_HUMAN			
um10430	32,0	13,3	1	CCD49_HUMAN			
um10439	22,3	11,5	1	KT3K_HUMAN			
um10447	35,3	21,7	1	H10_HUMAN			
um10448	70,0	52,0	1	AP1M1_HUMAN			
um10449	33,1	15,8	1	SFT2B_HUMAN	um10449.2	28,5	*U. maydis* gene model changed
um10455	30,3	11,2	1	HYEP_HUMAN	um01938	30,6	*U. maydis* gene model changed
um10469	70,5	60,3	1	RS5_HUMAN			
um10481	33,3	11,8	1	PARK7_HUMAN			
um10491	20,7	7,6	0	Q9UF83_HUMAN	um15060	23,5	BRH not in *U.maydis*-to-*H.sapiens* set
um10496	30,4	19,8	1	Q96BA2_HUMAN			
um10504	39,1	28,6	1	A5D8W6_HUMAN			
um10535	35,1	12,6	1	CWC15_HUMAN			
um10552	38,5	9,4	1	Q9UM02_HUMAN			
um10566	35,8	19,7	1	WIPF1_HUMAN			
um10599	41,9	30,0	1	Q9BQL0_HUMAN			
um10607	38,5	4,9	1	A4D109_HUMAN			
um10611	60,0	37,2	1	Q4W5J5_HUMAN			
um10613	43,4	26,5	1	CAB39_HUMAN			
um10628	21,4	4,9	1	Q6ZTM9_HUMAN			
um10639	23,6	13,1	1	A0PJL9_HUMAN			
um10642	44,7	26,6	1	A4D0W0_HUMAN			
um10643	27,8	13,2	1	PYRD_HUMAN			
um10665	25,5	8,2	0	ACADS_HUMAN	um01049	38,7	BRH is member of the *U.maydis*-to-*H.sapiens* set
um10666	40,5	30,0	1	DDX23_HUMAN			
um10674	28,4	15,5	1	RBMX2_HUMAN			
um10693	21,0	6,2	0	Q4G0Z0_HUMAN	um15060	23,5	BRH not in *U.maydis*-to-*H.sapiens* set
um10695	55,1	11,4	1	NDUS1_HUMAN			
um10698	24,0	13,9	1	NIP30_HUMAN			
um10723	25,9	13,7	1	EVA2_HUMAN			
um10732	29,9	14,1	1	Q9UQ10_HUMAN			
um10735	44,5	21,3	1	ODBA_HUMAN			
um10747	34,5	19,7	1	EXOS8_HUMAN			
um10750	21,0	4,9	1	CQ028_HUMAN			
um10754	26,2	4,9	1	ATP5E_HUMAN			
um10755	30,4	4,9	1	CQ061_HUMAN			
um10764	20,1	6,0	1	TRA16_HUMAN			
um10769	44,2	28,9	1	Q6FG22_HUMAN			
um10782	27,1	8,0	1	Q0VAX8_HUMAN			

um10783	48,1	21,8	1	Q59EL0_HUMAN			
um10796	36,4	11,3	1	XP_950979			
um10799	41,5	29,9	1	CHMP3_HUMAN			
um10807	66,6	55,6	1	PRP8_HUMAN			
um10810	73,0	62,4	1	PSDE_HUMAN			
um10811	48,7	32,6	1	ARL8A_HUMAN			
um10820	38,4	20,7	1	NDUA2_HUMAN			
um10825	45,5	22,0	1	HCD2_HUMAN			
um10847	34,9	4,9	1	Q53XX0_HUMAN			
um10862	34,2	23,7	1	UB2J1_HUMAN			
um10879	30,6	4,9	1	Q549C7_HUMAN			
um10892	43,6	33,3	1	Q9BY40_HUMAN			
um10901	64,9	39,4	1	Q5U5U6_HUMAN			
um10902	42,2	13,3	1	RBM8A_HUMAN			
um10910	25,0	9,2	1	RAD9A_HUMAN			
um10915	50,0	37,6	1	Q5SQH4_HUMAN			
um10917	75,0	49,6	1	RBX1_HUMAN			
um10919	29,4	19,3	1	A6NDW8_HUMAN	um15049	26,7	*U. maydis* gene model changed
um10929	20,8	5,3	1	Q96JY9_HUMAN			
um10933	30,0	12,6	1	NP_001071634	um15022	15,6	*U. maydis* gene model changed
um10935	21,7	10,2	0	AP3M1_HUMAN	um03144	30,5	BRH is member of the *U.maydis*-to-*H.sapiens* set
um10955	20,7	8,2	0	Q658P0_HUMAN	um03392.2		*U. maydis* gene model changed
um10961	42,2	30,6	1	XP_001134369			
um10988	66,4	55,8	1	AP2S1_HUMAN			
um10989	20,5	5,5	1	Q6SYC0_HUMAN			
um11015	20,3	4,9	0	NP_660205	um03372	23,6	BRH is member of the *U.maydis*-to-*H.sapiens* set
um11031	21,2	4,9	1	Q5SZP4_HUMAN			
um11032	21,2	4,9	1	Q5SZP4_HUMAN			
um11036	43,7	33,6	1	PLRG1_HUMAN			
um11038	54,7	8,6	1	Q7LD69_HUMAN			
um11043	44,5	29,6	1	SMD3_HUMAN			
um11045	27,7	13,6	1	Q9BRD3_HUMAN	um15008		*U. maydis* gene model changed
um11077	24,0	12,0	1	Q53FW5_HUMAN			
um11085	38,5	15,6	1	PPIL4_HUMAN			
um11107	27,2	12,2	1	NMRL1_HUMAN			
um11112	28,1	10,4	0	CHDH_HUMAN	um01872	32,1	BRH is member of the *U.maydis*-to-*H.sapiens* set
um11126	21,9	11,2	0	WDR5_HUMAN	um02856	27,7	BRH not in *U.maydis*-to-*H.sapiens* set
um11130	22,9	11,1	0	THEM2_HUMAN	um04666	23,4	BRH is member of the *U.maydis*-to-*H.sapiens* set
um11152	20,4	7,2	1	ACMSD_HUMAN			
um11157	23,9	9,6	1	SAKS1_HUMAN			
um11162	61,2	12,1	1	Q5VTW0_HUMAN			
um11170	61,0	7,7	1	NDUV1_HUMAN			
um11178	26,4	12,8	0	RU2B_HUMAN	um12111	28,1	BRH is member of the *U.maydis*-to-*H.sapiens* set
um11198	22,9	7,7	1	TERT_HUMAN			
um11204	49,3	29,3	1	A6NLB5_HUMAN			
um11214	23,9	13,8	1	Q86V49_HUMAN			

um11217	20,6	5,5	1	Q6ICN9_HUMAN			
um11247	31,5	17,2	1	Q5TGH1_HUMAN			
um11252	27,1	14,1	1	CSN4_HUMAN			
um11264	20,4	4,9	1	Q59EZ2_HUMAN	um15057		*U. maydis* gene model changed
um11283	35,1	19,2	1	Q9H5X4_HUMAN			
um11285	33,3	4,9	1	Q5VXS7_HUMAN			
um11289	25,8	9,4	1	LNP_HUMAN			
um11292	22,0	9,0	1	Q86TE5_HUMAN			
um11303	24,3	13,8	1	A4D1H8_HUMAN			
um11309	31,3	15,7	1	Q53HJ6_HUMAN			
um11318	48,0	37,0	1	ARD1B_HUMAN			
um11337	43,7	14,2	1	FAAA_HUMAN	um11337.2		*U. maydis* gene model changed
um11342	47,5	16,4	1	FA50A_HUMAN			
um11352	30,2	9,9	1	ASAH2_HUMAN			
um11362	21,4	4,9	1	A5LIN1_HUMAN			
um11364	37,7	14,8	1	Q8WV54_HUMAN			
um11368	34,7	23,3	1	NP_001905			
um11386	26,2	12,8	1	A6NED4_HUMAN			
um11395	21,4	10,6	1	Q5TGY03_HUMAN			
um11402	45,7	20,7	1	NP_940842			
um11405	47,0	26,7	1	APC11_HUMAN			
um11421	53,8	25,5	1	MMSA_HUMAN			
um11427	27,1	15,1	1	SYF2_HUMAN			
um11445	21,0	8,0	1	Q9P0D7_HUMAN			
um11447	26,4	11,8	1	TIP_HUMAN	um11447.2		*U. maydis* gene model changed
um11448	47,6	10,4	1	NDUS8_HUMAN			
um11451	31,0	18,3	1	CSN6_HUMAN			
um11452	27,3	4,9	1	Q5EE01_HUMAN			
um11463	34,8	20,1	1	DYHC_HUMAN	um15045		*U. maydis* gene model changed
um11473	38,9	16,6	1	UXS1_HUMAN	um02829		*U. maydis* gene model changed
um11482	61,8	43,1	1	UBC12_HUMAN			
um11483	48,9	11,3	1	PIMT_HUMAN			PIMT/PCMT1 (*H.sapiens* gene name)
um11521	27,6	13,7	1	Q5T411_HUMAN			
um11524	21,0	7,2	1	KR133_HUMAN			
um11525	84,2	61,5	1	PP2AA_HUMAN			
um11530	24,5	5,5	1	Q6ZV49_HUMAN			
um11539	37,5	27,1	1	SNF8_HUMAN			
um11540	26,9	11,5	0	Q53F46_HUMAN	um00182	28,5	BRH is member of the *U.maydis*-to-*H.sapiens* set
um11554	27,6	17,5	0	CHDH_HUMAN	um01872	32,1	BRH is member of the *U.maydis*-to-*H.sapiens* set
um11556	54,8	18,6	1	NP_004083			
um11557	43,3	29,8	1	CCD94_HUMAN			
um11561	34,0	15,6	1	RL28_HUMAN			
um11594	26,5	10,1	1	TMM66_HUMAN			
um11600	45,6	22,8	1	Q38G75_HUMAN			
um11619	51,2	40,0	1	Q7Z4W8_HUMAN			
um11632	22,6	11,2	1	Q6ZMF6_HUMAN			

um11636	29,9	15,3	1	VPS16_HUMAN			
um11649	24,4	11,1	0	Q5JXM5_HUMAN	um05703	26,3	BRH is member of the *U.maydis*-to-*H.sapiens* set
um11675	20,2	7,7	1	Q5NV75_HUMAN			
um11725	24,7	13,8	1	Q1HE27_HUMAN			
um11726	29,2	14,7	1	MPPD1_HUMAN			
um11731	24,2	4,9	1	NDUA3_HUMAN			
um11758	30,9	19,1	1	CC124_HUMAN			
um11770	30,2	16,3	1	Q9BRZ8_HUMAN			
um11771	31,0	19,5	1	ALG14_HUMAN			
um11795	25,3	6,4	1	Q5VV89_HUMAN			
um11835	22,5	6,5	1	Q6ZW28_HUMAN			
um11838	21,9	11,7	1	S39A9_HUMAN	um15030		*U. maydis* gene model changed
um11854	33,6	22,8	1	ANM5_HUMAN	um15057		*U. maydis* gene model changed
um11862	37,0	4,9	1	Q9HB72_HUMAN			
um11866	41,0	22,9	1	A0MNP2_HUMAN			
um11882	62,7	43,4	1	PPIL1_HUMAN			
um11896	42,1	10,6	1	NDUS3_HUMAN			
um11898	21,4	4,9	1	Q8IW09_HUMAN	um01684		*U. maydis* gene model changed
um11901	28,5	15,4	1	Q9Y392_HUMAN			
um11905	42,4	20,1	1	NP_065994			
um11912	38,5	21,2	1	Q5TAY8_HUMAN			
um11932	52,9	10,0	1	MCCC2_HUMAN			
um11938	33,6	4,9	1	NP_001007101			
um11948	68,5	54,8	1	UBL5_HUMAN			
um11952	56,3	29,0	1	HS90B_HUMAN			
um11966	38,0	26,6	1	CUL3_HUMAN	um11966.2		*U. maydis* gene model changed
um11983	70,2	53,5	1	PHF5A_HUMAN			
um11999	52,5	34,6	1	RBBP4_HUMAN			
um12006	40,3	19,6	1	SIRT5_HUMAN			
um12021	42,3	6,1	1	ISOC1_HUMAN			
um12025	21,7	9,7	0	XP_001128001	um01179	21,9	BRH not in *U.maydis*-to-*H.sapiens* set
um12034	77,8	58,9	1	NEDD8_HUMAN			
um12047	25,5	15,1	1	Q9NPA8_HUMAN			
um12051	27,4	12,9	0	CSAD_HUMAN	um02125	31,3	BRH is member of the *U.maydis*-to-*H.sapiens* set
um12058	23,0	9,5	0	A6NP33_HUMAN	um00982	31,4	BRH is member of the *U.maydis*-to-*H.sapiens* set
um12059	23,1	7,6	1	Q5VZU3_HUMAN	um15007	19,6	*U. maydis* gene model changed
um12072	38,4	27,1	1	MP2K6_HUMAN	um15092		*U. maydis* gene model changed
um12084	33,3	4,9	1	MOS2S_HUMAN			
um12085	33,2	19,1	1	CK073_HUMAN			
um12092	22,5	4,9	0	Q6N065_HUMAN			
um12097	37,3	23,4	1	NP_001001660			
um12106	35,5	6,7	1	Q5VZU8_HUMAN			
um12111	29,9	18,8	1	RU2B_HUMAN			
um12117	20,7	4,9	1	XP_001130275			
um12133	20,7	4,9	1	A0N0Q1_HUMAN			
um12165	29,4	9,2	1	NP_115742			

um12169	23,1	11,5	1	Q9HAS3_HUMAN			
um12178	20,3	8,5	0	A4D2K9_HUMAN	um03892	38,9	BRH not in *U.maydis* -to-*H.sapiens* set
um12224	20,6	9,9	1	TFP11_HUMAN			
um12234	28,6	18,3	1	ATG12_HUMAN			
um12243	32,9	17,3	1	NP_620706			
um12244	43,7	25,5	1	Q6IB35_HUMAN			
um12253	20,9	8,6	1	Q8ND10_HUMAN			
um12269	34,8	19,3	1	HAOX1_HUMAN			
um12278	47,3	34,5	1	DYL1_HUMAN			
um12282	28,3	5,6	1	IF3C_HUMAN			
um12290	25,0	13,8	1	Q9NTQ6_HUMAN			
um12299	35,4	10,3	1	GLOD5_HUMAN			
um12300	26,7	15,3	0	MCCA_HUMAN	um04382	43,8	BRH is member of the *U.maydis* -to-*H.sapiens* set
um12305	41,0	22,3	1	GTPB1_HUMAN			
um12323	20,6	4,9	1	Q15BH2_HUMAN			
um12327	36,4	8,1	1	GEPH_HUMAN			
um12337	30,5	14	1	NP_001032248			
um12341	24,3	9,0	0	Q5TZW8_HUMAN	um01424	26,1	BRH not in *U.maydis* -to-*H.sapiens* set

U.maydis -to-_H.sapiens_ proteins found additionally by domain analysis

***U.maydis* code**	**%identity to *H.sapiens***	**%identity to *S.cerevisiae***	**BBH**	***H.sapiens* code**	**BRH *U.maydis* cod**	**% identity to BRH**	**comments**
um00206	21,5	8,5	0	SVOP_HUMAN	um02900	23,4	BRH not in *U.maydis* -to-*H.sapiens* set
um00394	21,2	9,2	0	A6NFJ2_HUMAN	um05719	23,1	BRH is member of the *U.maydis* -to-*H.sapiens* set
um00499	20,5	9,2	1	A5D6V5_HUMAN			
um00687	21,4	11,1	1	WDR23_HUMAN			
um01335	54,1	8,7	1	GCDH_HUMAN			GCDH (*H.sapiens* gene name)
um01523	22,3	9,2	1	FOXI1_HUMAN			
um01682	25,1	11,0	1	BCAS2_HUMAN			
um01938	30,8	6,2	1	HYEP_HUMAN			EPHX1 (*H.sapiens* gene name)+H30
um01965	22,9	10,5	1	Q6FI62_HUMAN			
um02327	20,4	7,7	1	U432_HUMAN			
um02482	25,2	13,4	1	WDR70_HUMAN			
um03036	22,9	12,4	1	WDR55_HUMAN			
um03234	26,8	16,4	1	Q5JR99_HUMAN	um03234.2		*U. maydis* gene model changed
um03625	22,2	7,1	0	Q96KB7_HUMAN	um15087	14,3	*U. maydis* gene model changed
um04203	20,7	5,4	1	PKHQ1_HUMAN			
um04247	23,5	9,4	0	CHLE_HUMAN	um11540	26,7	BRH is member of the *U.maydis* -to-*H.sapiens* set
um05084	23,4	6,1	1	Q5T9U3_HUMAN			
um05173	27,7	9,1	1	DNJC3_HUMAN			
um05267	20,9	8,1	1	Q6UY23_HUMAN			
um05686	20,2	8,5	1	NP_937847			
um10348	20,9	8,7	1	Q6WG76_HUMAN			
um10898	33,5	21,9	1	DHR10_HUMAN			
um10952	38,0	8,0	1	Q9BV39_HUMAN	um15032	8,6	*U. maydis* gene model changed
um11344	20,7	10,6	1	A2A3C9_HUMAN			
um11505	23,8	5,0	1	MYPT2_HUMAN			

um11517	28,0	6,0	1	NDUA5_HUMAN			
um11788	28,8	18,5	1	SIRT4_HUMAN	um05239		*U. maydis* gene model changed
um11861	23,6	7,7	1	Q8NAM5_HUMAN	um15036	22,6	*U. maydis* gene model changed
um11972	21,0	5,6	1	Q8ZNS9_HUMAN	um15070	12,9	*U. maydis* gene model changed
um12121	21,8	4,0	1	A1A520_HUMAN			
**681 proteins**			**620**	**91% with Best Bidirectional Hit**			

List of alternative Best Rezipropal Hits of the *U.maydis-* to-*H.sapiens* set

**BRH *U. maydis* cod**	**amount**	**relation to the *U.maydis* -to-*H.sapiens* set**
um00005	1	BRH not in *U.maydis* -to-*H.sapiens* set
um00182	1	BRH is member of the *U.maydis* -to-*H.sapiens* set
um00982	1	BRH not in *U.maydis* -to-*H.sapiens* set
um00984	1	BRH is member of the *U.maydis* -to-*H.sapiens* set
um01049	3	BRH is member of the *U.maydis* -to-*H.sapiens* set
um01099	1	BRH is member of the *U.maydis* -to-*H.sapiens* set
um01179	2	BRH not in *U.maydis* -to-*H.sapiens* set
um01335	2	BRH is member of the *U.maydis* -to-*H.sapiens* set
um01382	1	BRH not in *U.maydis* -to-*H.sapiens* set
um01424	1	BRH not in *U.maydis* -to-*H.sapiens* set
um01872	6	BRH is member of the *U.maydis* -to-*H.sapiens* set
um01935	1	BRH is member of the *U.maydis* -to-*H.sapiens* set
um01938	1	BRH is member of the *U.maydis* -to-*H.sapiens* set
um02125	1	BRH is member of the *U.maydis* -to-*H.sapiens* set
um02856	1	BRH not in *U.maydis* -to-*H.sapiens* set
um02900	1	BRH not in *U.maydis* -to-*H.sapiens* set
um02993	1	BRH is member of the *U.maydis* -to-*H.sapiens* set
um03144	1	BRH is member of the *U.maydis* -to-*H.sapiens* set
um03372	1	BRH is member of the *U.maydis* -to-*H.sapiens* set
um03392.2	1	BRH not in *U.maydis* -to-*H.sapiens* set
um03662	1	BRH not in *U.maydis* -to-*H.sapiens* set
um03910	1	BRH is member of the *U.maydis* -to-*H.sapiens* set
um04061	1	BRH is member of the *U.maydis* -to-*H.sapiens* set
um04382	1	BRH is member of the *U.maydis* -to-*H.sapiens* set
um04666	1	BRH is member of the *U.maydis* -to-*H.sapiens* set
um04677	1	BRH is member of the *U.maydis* -to-*H.sapiens* set
um05131	1	BRH is member of the *U.maydis* -to-*H.sapiens* set
um05240	1	BRH not in *U.maydis* -to-*H.sapiens* set
um05703	1	BRH is member of the *U.maydis* -to-*H.sapiens* set
um05719	1	BRH is member of the *U.maydis* -to-*H.sapiens* set
um05773	1	BRH is member of the *U.maydis* -to-*H.sapiens* set
um05820	1	BRH not in *U.maydis* -to-*H.sapiens* set
um06071	2	BRH is member of the *U.maydis* -to-*H.sapiens* set
um10149	1	BRH is member of the *U.maydis* -to-*H.sapiens* set
um10177	1	BRH is member of the *U.maydis* -to-*H.sapiens* set
um10898	1	BRH not in *U.maydis* -to-*H.sapiens* set
um11337.2	1	BRH not in *U.maydis* -to-*H.sapiens* set

um11470	1	BRH not in *U.maydis* -to-*H.sapiens* set
um11540	2	BRH is member of the *U.maydis* -to-*H.sapiens* set
um11556	2	BRH is member of the *U.maydis* -to-*H.sapiens* set
um11682	1	BRH not in *U.maydis* -to-*H.sapiens* set
um11932	1	BRH is member of the *U.maydis* -to-*H.sapiens* set
um11983	1	BRH is member of the *U.maydis* -to-*H.sapiens* set
um12111	1	BRH is member of the *U.maydis* -to-*H.sapiens* set
um15000.2	1	BRH not in *U.maydis* -to-*H.sapiens* set
um15060	3	BRH not in *U.maydis* -to-*H.sapiens* set
